# Gender Bias in Letters of Recommendation for Cardiothoracic Surgery Applicants

**DOI:** 10.1016/j.atssr.2023.07.007

**Published:** 2023-08-09

**Authors:** Hope A. Feldman, Marianna V. Papageorge, Nathaniel Deboever, Heath Goodrum, Samuel Camp, Shanda Blackmon, Jennifer S. Lawton, Mara B. Antonoff

**Affiliations:** 1 Division of Surgery, Department of Thoracic and Cardiovascular Surgery, The University of Texas MD Anderson Cancer Center, Houston, Texas; 2 Department of Surgery, Yale University School of Medicine, New Haven, Connecticut; 3 Enterprise Development and Integration, MD Anderson Cancer Center, Houston, Texas; 4 Division of General Thoracic Surgery, Mayo Clinic, Rochester, Minnesota; 5 Division of Cardiac Surgery, Department of Surgery, Johns Hopkins University School of Medicine, Baltimore, Maryland

## Abstract

**Background:**

Bias in letters of recommendation may have an impact on assessment of the cardiothoracic surgical applicant. Letters written for fellowship candidates might differ on the basis of the applicant's gender, although prior investigations have not explored this population. We aimed to evaluate gender differences in letters of recommendation written for cardiothoracic surgery applicants.

**Methods:**

Letters of recommendation written for applicants between 2015 and 2020 to a large, 2-year academic cardiothoracic surgical training program were reviewed. Data regarding applicants and letter writers were captured. A library of terms to describe applicants was created. Natural language processing was employed to detect differences in use of these terms in letters written for men vs letters written for women. Letter content was compared across applicant and writer characteristics by Kruskal-Wallis and *t*-tests.

**Results:**

There were 1707 letters of recommendation for 553 applicants analyzed: 430 letters for 147 (26.6%) women and 1277 letters for 406 (73.4%) men. Mean letter length was 498.4 (SD 209.3) words. Letters for men were longer (518.2 vs 491.7 words; *P =* .022). Men were more often described with terms of humility (*P <* .01), professionalism (*P <* .01), and integrity/honesty (*P <* .01), whereas women were more frequently described by terms of family (*P <* .01) and leadership (*P <* .01). Men writers described men applicants more often with gendered language (*P <* .01) and terms of humility (*P <* .01), personality (*P <* .01), and integrity/honesty (*P <* .01).

**Conclusions:**

We identified gender-based differences in letters of recommendation for cardiothoracic surgery applicants as well as differences based on the writer's gender. These findings are critical to consider in the assessment of applicants as efforts are needed to minimize bias and to support a diverse future of our specialty’s workforce.

**In Short**

▪ There are substantial differences in the terms used in letters of recommendation for women vs men cardiothoracic surgery applicants.
▪ There exist differences in the language used by women and men writers for women and men applicants.
▪ Women applicants request letters from women faculty at a higher frequency than their male counterparts do.

Letters of recommendation serve as a valuable component of applications to residency and fellowship, often highlighted as one of the most important factors in selecting which candidates to interview. Prior studies have shown that in various surgical subspecialties, letter writers will emphasize different traits based on the applicant's gender, with women described by communal (relationship-based) terms that emphasize likeability and teamwork, whereas men were praised by agentic (achievement-based) terms that emphasize leadership.^1^

There remains a disparity in representation of women in the field of cardiothoracic surgery at every level of training and career progression.^2^ Work is ongoing to help understand these disparities and to mitigate the biases that may adversely affect women in this field.^3^

Whereas gender-based differences in letters of recommendation have been well described in other fields, no studies have yet explored the bias in letters of recommendation for the cardiothoracic surgery applicant population. We sought to use natural language processing (NLP) to review letters of recommendation for applicants to this field.

## Material and Methods

### Data Sources

We retrospectively analyzed letters of recommendation written for individuals who applied to a traditional-track, 2-year cardiothoracic surgery training program from 2015 to 2020. The academic institution to which these applicants applied consistently receives applications from 72% of match participants nationally.^4^ Letters of recommendation were converted to portable document format (PDF) with Adobe Acrobat, and all elements of applicant-identifying information were removed. Repeat applicants were counted only once in the year in which they initially applied. Applicants outside of North America were excluded.

We recorded the following applicant information: gender; American Board of Surgery In-Training Examination scores of postgraduate years 1 to 3, with the highest score taken if more than 1 score was available for a particular training year; and research productivity, calculated by summing the number of peer-reviewed publications, poster presentations, oral presentations, and book chapters. We recorded the following letter writer information: gender, academic rank, and geographic region (Supplemental Table 1). If these elements were not apparent from the application, internet searches were performed.

### Letter of Recommendation Analysis

The text of each letter was analyzed by NLP, a set of techniques used to create data sets from text to be evaluated by statistical models. Text was extracted from the PDFs using Tesseract and deidentified using NLM text scrubber. Text analysis was performed using Python, including the NLTK, SciPy stats, and statsmodels libraries. The entire content of the letter, including word count, was processed and recorded. We then developed a library of terms based on prior publications in the field and recorded the frequency of each category (Supplemental Table 2).^5^ These groups included both agentic terms, which more often describe men, and communal terms, which more often describe women.

### Statistical Analysis

Letter content was compared across both applicant and writer characteristics with *t*-tests and Kruskal-Wallis tests. Statistical analyses were conducted by SAS software (SAS Institute) with significance set at < .05.

## Results

We analyzed 1707 letters of recommendation for 553 applicants: 430 letters for 147 (26.6%) women applicants and 1277 letters for 406 (73.4%) men applicants (Table 1). Across postgraduate years 1 to 3, mean in-service examination score percentiles were higher for men (61; SD 29) vs women (51; SD 21; *P <* .01), with no difference in mean research productivity (*P =* .2). Letters consisted of a mean word count of 498.4 (SD 209.3), with letters for men longer than those for women (men, 518.2 words; women, 491.7 words; *P =* .02). Most letter writers were men (89.8%), and this held true for both women (85.8%) and men (91.1%) applicants (Supplemental Table 3). Interestingly, women writers wrote letters of recommendation for women applicants at higher proportions than men writers (14.2% vs 8.9%; *P <* .01).

**Table 1:** Description of Applicants and Letters of Recommendation

Variable	All Applicants (N = 553)	Women (n = 147)	Men (n = 406)	*P* Value
ABSITE percentile	58 (27)	51 (21)	61 (29)	<.01
Research productivity	16 (18)	16 (18)	16 (18)	.2
Word count per letter of recommendation	498.4 (209.3)	491.7 (205.1)	518.2 (220.4)	.02

Values are reported as mean (SD).

ABSITE, American Board of Surgery In-Training Examination.

Regarding academic rank, most writers were assistant professors (61.2%). Women writers represented 10.7% of assistant professors, 7.0% of nonacademic or unknown rank, and 16.8% of associate or full professors. Letter writer rank was similar compared across women and men applicants.

Men applicants were more often described with themes of humility (*P <* .01), professionalism (*P <* .01), and integrity/honesty (*P <* .01) and with the use of gendered language (*P <* .01). In contrast, women were more often described by terms of leadership (*P =* .04; Supplemental Table 4). Further breakdown of individual words demonstrated statistically significant differences in family roles, personality traits, and accomplishments based on gender (Supplemental Table 5). Women applicants had the term husband (*P <* .01) and single (*P =* .03) included more often than men applicants. Similarly, women applicants were more often described with terms like introversion (*P <* .01), sympathetic (*P =* .02), passive (*P =* .02), cheer (*P =* .04), care (*P =* .04), and compassion (*P =* .05). They were also described more often by leadership (*P <* .01), mastery (*P <* .01), mentorship (*P <* .01), ability (*P =* .02), and scholarship (*P =* .02). Men applicants were more often described with terms including professional (*P <* .01), serious (*P <* .01), nice (*P =* .02), and humble (*P =* .02) and with reference to fund of knowledge (*P =* .05).

In evaluating letters stratified by letter writer gender, women writers used the themes of agency (*P =* .03), grindstone (*P =* .04), leadership (*P <* .01), research (*P =* .02), respect (*P <* .01), take charge (*P <* .01), teaching (*P <* .01), and teamwork (0=0.01) more often than men writers (Supplemental Table 6). In contrast, men writers more often wrote about future promise, but this did not meet statistical significance (*P =* .5). Otherwise, there were no differences in themes used.

On further stratifying by letter writer gender and applicant gender, men writers described men applicants more often with gendered language (*P <* .01) and terms related to humility (*P <* .01), personality (*P <* .01), and integrity/honesty (*P <* .01). Women writers commented on clinical knowledge in men applicants (*P =* .04) and take-charge themes in women applicants (*P =* .03; Table 2).

**Table 2:** Letter Theme by Letter Writer Gender, Stratified by Applicant Gender

Variable	Women Writers (n = 175)			Men Writers (n = 1532)		
	Women Applicants (n = 61)	Men Applicants (n = 114)	*P* Value	Women Applicants (n = 369)	Men Applicants (n = 1163)	*P* Value
Agency	4.4 (3.6)	4.0 (2.6)	.8	3.8 (2.9)	3.6 (2.7)	.2
Communality	3.3 (2.2)	3.2 (2.2)	.8	3.1 (2.5)	3.0 (2.3)	.9
Academic background, awards, scholarship	2.2 (3.3)	2.5 (3.6)	.3	2.1 (3.4)	2.1 (3.1)	.9
Aptitude, intelligence	1.0 (2.0)	1.2 (1.7)	.4	0.8 (1.3)	0.8 (1.3)	.6
Clinical skills, fund of knowledge	0.4 (0.8)	0.7 (0.9)	.04	0.5 (0.8)	0.5 (0.8)	.3
Communication	0.2 (0.7)	0.1 (0.4)	.3	0.1 (0.4)	0.1 (0.4)	1.0
Confidence	0.5 (0.8)	0.4 (0.6)	.6	0.4 (0.8)	0.4 (0.7)	.7
Family	0.6 (1.5)	0.5 (1.0)	.4	0.6 (1.3)	0.5 (1.2)	.4
Hobbies	0.02 (0.1)	0.06 (0.3)	.5	0.03 (0.2)	0.02 (0.2)	.6
Future promise	0.05 (0.2)	0.04 (0.2)	.7	0.05 (0.2)	0.06 (0.3)	.5
Gendered language	0.2 (0.5)	0.2 (0.5)	.9	0.1 (0.3)	0.2 (0.5)	<.01
Grindstone	2.0 (2.1)	2.1 (1.9)	.5	1.9 (1.7)	1.8 (1.7)	.2
Humble	0.08 (0.3)	0.1 (0.4)	.2	0.05 (0.2)	0.1 (0.3)	<.01
Leadership	0.9 (1.5)	0.9 (1.0)	.3	0.7 (1.2)	0.6 (1.0)	.1
Likeability	0.4 (0.5)	0.3 (0.5)	.4	0.2 (0.4)	0.2 (0.5)	.1
Maturity, emotional intelligence	0.3 (0.6)	0.3 (0.5)	.5	0.2 (0.5)	0.2 (0.5)	.3
Physical description	0.07 (0.4)	0.05 (0.3)	.8	0.03 (0.3)	0.05 (0.3)	.3
Professionalism	0.3 (0.7)	0.3 (0.5)	.3	0.2 (0.6)	0.3 (0.7)	.01
Research	3.8 (4.0)	4.1 (4.9)	1.0	3.2 (3.6)	3.1 (3.8)	.5
Respect	0.5 (0.8)	0.4 (0.7)	.2	0.2 (0.4)	0.2 (0.5)	.1
Retention, recruitment	0 (0)	0.009 (0.09)	.5	0.005 (0.07)	0.003 (0.06)	.6
Personality	0.5 (0.7)	0.5 (0.8)	.9	0.3 (0.7)	0.5 (0.8)	<.01
Superlatives	3.6 (2.6)	3.7 (2.9)	.8	3.5 (2.5)	3.5 (2.8)	.6
Take charge	0.2 (0.4)	0.08 (0.3)	.03	0.06 (0.2)	0.06 (0.2)	1.0
Teaching	1.1 (1.1)	1.1 (1.3)	.4	0.8 (1.1)	0.8 (1.2)	.2
Teamwork	0.7 (1.3)	0.9 (1.0)	.2	0.6 (1.1)	0.7 (1.1)	.4
Technical ability/skills	0.7 (0.7)	0.6 (1.0)	.2	0.7 (0.8)	0.6 (0.8)	.3
Trustworthy	0.1 (0.3)	0.1 (0.4)	1.0	0.1 (0.3)	0.1 (0.3)	.3
Integrity, honesty	0.2 (0.4)	0.2 (0.5)	.8	0.1 (0.4)	0.2 (0.6)	<.01
Work ethic	0.4 (0.6)	0.4 (0.6)	.3	0.3 (0.6)	0.4 (0.6)	.1

Values are reported as mean (SD).

## Comment

In this study, we sought to objectively evaluate the language used to describe the men and women applicants to cardiothoracic surgical training and to explore the ways in which gender bias may result in different qualities and accomplishments being emphasized by letter writers. We found that letters written for men were longer than those written for women and that they more often used terms that emphasized humility, professionalism, and integrity. Letters for women applicants more often emphasized leadership, mastery, ability, and scholarship.

The findings from our NLP review of cardiothoracic surgical applicants stand in contrast to those of other surgical subspecialties. We found that the traits emphasized in men and women applicants were reliably and consistently different. A review of applications to pediatric surgery fellowship or complex general surgical oncology fellowship did not show any meaningful difference in letters of recommendation based on gender. Interestingly, 50% of fellows in pediatric surgery and 33% in complex general surgical oncology are women.^6^^,^^7^ It is possible that in fields that have more women faculty and trainees, this exposure is protective against bias.

Recruitment of qualified candidates to the field of cardiothoracic surgery is imperative. There have been several obstacles, including implicit biases held by both trainees and mentors, that have made entry into the field exceedingly challenging for minority groups.^8^ Finding that different attributes are emphasized according to the gender of the applicant and that of the writer highlights a potential area for improvement in the application process. In addition, awareness about historical biases may enable recommendation letter authors to reevaluate their own writing in the future.

One of the more important findings of our study was that although only 10% of letter writers were women, female faculty wrote 14% of letters for women applicants. On several occasions, it was apparent that women applicants obtained letters from women cardiothoracic surgeons outside of their home institution. To that end, social media has emerged as an important tool that has enabled women seeking gender-concordant mentorship in cardiothoracic surgery to find faculty mentors.^9^ This finding highlights the importance of having gender-concordant mentors available, which in a field where only 17% of faculty are women is undoubtedly a challenge.

Whereas our study aimed to use a quantitative and therefore unbiased approach, there are limitations inherent in the design. First, our study included a single institution during a finite period and captured only a portion of cardiothoracic surgery applicants, which may introduce potential bias related to generalizability and sample size. Second, we did not account for complex characteristics of the authors, including letters written by the same author or authors from the same institution. In addition, we included only women and men in our study and recommend that future studies include nonbinary writers and applicants as is applicable. Similarly, other applicant demographics, such as race, are no longer included in the formal applications but may have affected results. Finally, we are not able to determine any potential impact of these differences in letters on candidate selection as such downstream end points extend beyond the scope of outcomes of interest for this study. However, we anticipate that this work sets a foundation for future scholarship, identifying additional knowledge gaps and areas for further investigation.

In this study, we reviewed 1707 letters of recommendation with NLP to evaluate for discrepancies in the language used to describe men compared with women applicants. We found that different attributes were highlighted on the basis of the applicant's gender. In addition, it seems that women writers used similar language when describing men and women applicants. These findings are critical to consider as we conduct assessment of applicants in cardiothoracic surgery and highlight the need for educational and standardized practices. Moreover, ongoing efforts to optimize candidate selection are in strong need to minimize bias and to support a diverse workforce.
