# Supplementary material for: Gender Bias in Letters of Recommendation for Cardiothoracic Surgery Applicants
Source: Ann Thorac Surg Short Rep. 2023 Aug 9;1(4):696–700. doi: 10.1016/j.atssr.2023.07.007 (PMC11708520; doi:10.1016/j.atssr.2023.07.007)
Supplement: Supplementary Tables [file mmc1.docx]

Supplemental Table 1: Geographic regions

| Region | States |
| --- | --- |
| Northeast | CT, ME, MD, MA, NH, NJ, NY, PA, RI, VT |
| Southeast | AL, AR, DC, DE, FL, GA, KY, LA, MS, NC, SC, TN, VA |
| Southwest | AZ, NM, OK, TX |
| Midwest | IL, IN, IA, KS, MI, MN, MO, NE, ND, OH, WI |
| West | CA, CO, HI, NV, OR, UT, WA, WY |
| Canada |  |
| Puerto Rico |  |

Supplemental Table 2: Library of terms for analysis

| Category | Words |
| --- | --- |
| Agency | Achieve, achieving, achievement; Aggressive; Ambition, ambitious; Analytical, analytic; Aspiration, aspirational; Assertive; Attention; Autonomous, autonomy; Competent, competence; Competing, competitive, competition; Coordinator; Confident, confidence; Courage, courageous; Daring; Decisive; Dedicated, dedicating, dedicate; Defend, defending; Desire; Determination, determined; Dominant, dominating, dominated; Envy, enviable; Force, forceful, forcing; Goal oriented; Hard work, hard working, hard-work, hard-working, hard worker, hard-worker; Independent, independence; Industrious, industriously; Initiative, initiate, initiator; Intelligent, intelligence, intellectual; Lead, leader, leading, leadership; Master, mastered, mastery; Outspoken; pro-active, proactive; Problem-solver, problem solver; Rewards; Self direct, self direct, self-direct, self-directing, self directed, self-directed; Self motivated, self motivating, self-motivated, self-motivating; Self starter, self-starter, self starting, self-starting; Self direct, self-direct, self directing, self-directing, self directed, self-directed; Self driven, self-driven; Self reliant, self-reliant ; Serious; Skill, skilled, skillful; Strong; Take charge, take-charge; Tough; Under pressure; Work ethic, work-ethic |
| Communality | Admire, admired, admirable; Affection, affectionate; Agreeable; Amiable; Care, caring; Cheerful; colleague; Compassion, compassionate; Concern, concerned; Considerate; Cooperative, cooperating; Devoted, devoting; Eager; Easy to work; Emotional; Expressive; Feeling; Friend, friendly, friendship; Gentle; Good-nature, good nature, good-natured, good natured; Gullible; Happy; Help, helpful, helping; Interpersonal; Kind; Liked, likable, well-liked, well liked; Modest; Nurture, nurturing, nurtured; Passive; Sensitive; Soft; Soft spoken, soft-spoken; Sooth, soothing, soothed; Sympathy, sympathetic; Tactful, tact; team, team player, team-player, teamwork, team-work, team work; Tender; Timid; To other; Understand, understanding, understandable; Warm; Yield, yielding, yielded; respected; respect |
| Academic background, Awards, Scholarship | College; Masters Degree; Degree; Cum Laude; Magna Cum Laude; Summa Cum Laude; GPA, grade point average; STEP; honor, honors; scholarships, scholar; award; Alpha Omega Alpha, AOA |
| Aptitude/Intelligence | Able, ability; Adept; adroit; analytical; aptitude; brain; bright; capable; capacity; clever; competent; creative; expert; flair; genius; gift, gifted; inherent; instinct; insight, insightful; innate; intelligent, intelligence; natural; proficiency, proficient; propensity; skill, skilled, skillful; smart; talent, talented |
| Clinical skills, Fund of Knowledge | Clinical skills; patient care; physical exam; diagnostic, diagnosis; rounding, rounds; clinical knowledge; fund of knowledge; well-read |
| Communication | Communicate, communication, communicator |
| Confidence | Confident, confidence |
| Doubt raiser | Doubt; concern; uncertain; red flag |
| Family | Family; spouse; husband; wife; single; child, children, kids, kid; mother; father |
| Hobbies | hobby, hobbies; personal life; athlete; sport, sports; plays, play; outside of the hospital; cooking |
| Future promise | Future promise, promising; bright future; future leader; star in our field, star in the field |
| Gendered language | Man, woman, male, female, gentleman, lady |
| Grindstone | Hard working; Conscientious; Dependable; Meticulous; Thorough; Diligent; Dedicated; Careful; Reliable; Effort; Assiduous; Trust; Responsible; Methodical; Industrious; Busy; Work; Persist; Organized; Disciplined |
| Humble | Humble, humility, modest |
| Leadership | Lead, leader, leading, leadership |
| Likeability | agreeable, amiable, cheerful, considerate, cooperative, cooperating, friendly, good-nature, good-nature, good-natured, good natured, liked, likable, nice, well-liked, well liked |
| Maturity, Emotional intelligence | Mature, maturity, emotional intelligence |
| Physical description | Hair; height; weight; eyes; smile; dress; outfit; suit; tie |
| Professionalism | Professional, professionalism |
| Research | Research; publication, publications; presentations; publish; manuscript, manuscripts; journal, journals; grants, grant; project; fund; data; science; experiment; result, results; finding, findings; contribution, contributions, contributing; scholarly activity |
| Respect | Admire, admiration, respect, respected |
| Retention/recruitment | Recruit to our program, recruiting to our program; keep him/her; bring him/her back; hope he/she stays |
| Personality | Personality, personality trait, personality traits; introvert, introverted; extrovert, extroverted ; humor; funny; happy |
| Superlatives | Notable; Superior; Excellent; Superb; Outstanding; Unique; Exceptional; Unparalleled; Most; Wonderful; Terrific; Fabulous; Magnificent; Remarkable; Extraordinary; Amazing; Supreme; Best; Incredible; Standout; Exemplary; Brilliant |
| Take charge | self direct, self-direct, self-directing, self directed, self-directed; Self motivated, self motivating, self-motivated, self-motivating; Self starter, self-starter, self starting, self-starting; self driven, self-driven; take charge, take-charge, problem solver, problem-solver, proactive, pro-active, pro active |
| Teaching | Teaching, teacher; education, educator, educating; Instructor, instructing; supervise, supervisor; lecture, lecturer, lectured; mentor; adviser |
| Teamwork | team, team player, team-player, teamwork, team-work, team work |
| Technical Ability | Technical or Technically +: able, ability; adept; adroit; aptitude; bright; capable; capacity; clever; competent; creative; expert; flair; genius; gifted; inherent; instinct, instinctive; insight, insightful; innate; natural; proficiency, proficient; propensity; smart; talent, talented |
| Technical skills | Technical, technical skills, technically skilled, technically skillful; surgical skills, surgically skilled; spatial; hand eye coordination; dexterity; suture, suturing; knot, knots, knot tying |
| Trustworthy | trust; trustworthy |
| Integrity, Honesty | ethical, honest, honesty, integrity, moral |
| Work ethic | Goal oriented; Hard work, hard working, hard-work, hard-working, hard worker, hard-worker; work ethic, work-ethic |

Supplemental Table 3. Description of letter writers

| **Variable** | **All letters, n=1707** | **Letters for women, n=430** | **Letters for men, n=1277** | **p-value** |
| --- | --- | --- | --- | --- |
| **Letter Writer Gender** |  |  |  | 0.0019 |
| Woman | 175 (10.3%) | 61 (14.2%) | 14 (8.9%) |  |
| Man | 1532 (89.8%) | 369 (85.8%) | 1163 (91.1%) |  |
| **Letter writer rank** |  |  |  | 0.0813 |
| Non-academic, Unknown | 489 (28.7%) | 109 (25.4%) | 380 (29.8%) |  |
| Junior (Assistant) | 173 (61.2%) | 268 (62.3%) | 777 (60.9%) |  |
| Senior (Associate, Professor) | 1045 (10.1%) | 53 (12.3%) | 120 (9.4%) |  |
| **Letter writer region** |  |  |  | 0.0815 |
| Northeast | 626 (36.7%) | 144 (33.5%) | 482 (37.7%) |  |
| Southeast | 340 (19.9%) | 78 (18.1%) | 262 (20.5%) |  |
| Southwest | 185 (10.8%) | 53 (12.3%) | 132 (10.3%) |  |
| Midwest | 346 (20.3%) | 87 (20.2%) | 259 (20.3%) |  |
| West | 169 (9.9%) | 58 (13.5%) | 111 (8.7%) |  |
| Canada | 30 (1.8%) | 7 (1.6%) | 23 (1.8%) |  |
| Puerto Rico | 11 (0.6%) | 3 (0.7%) | 8 (0.6%) |  |

Supplemental Table 4. Mean counts of each theme, by applicant gender

| **Theme** | **Overall avg** | **Overall STD** | **Female avg** | **Female STD** | **Male avg** | **Male STD** | **p-value** |
| --- | --- | --- | --- | --- | --- | --- | --- |
| Agency | 3.707 | 2.791 | 3.91 | 2.959 | 3.64 | 2.729 | 0.074 |
| Communality | 3.077 | 2.354 | 3.14 | 2.442 | 3.06 | 2.324 | 0.523 |
| Technical Ability | 1.83 | 2.162 | 1.97 | 2.314 | 1.78 | 2.107 | 0.117 |
| Intelligence/Aptitude | 0.482 | 0.766 | 0.46 | 0.77 | 0.49 | 0.764 | 0.451 |
| Academic Background/Awards/Scholarship | 1.232 | 1.818 | 1.24 | 1.899 | 1.23 | 1.79 | 0.913 |
| Clinical Skills/Fund of Knowledge | 0.512 | 0.782 | 0.53 | 0.803 | 0.51 | 0.775 | 0.661 |
| Communication | 0.125 | 0.384 | 0.14 | 0.454 | 0.12 | 0.358 | 0.235 |
| Confidence | 0.401 | 0.677 | 0.42 | 0.758 | 0.39 | 0.648 | 0.419 |
| Family | 0.36 | 0.828 | 0.38 | 0.808 | 0.35 | 0.835 | 0.649 |
| Hobbies | 0.027 | 0.211 | 0.03 | 0.196 | 0.03 | 0.215 | 0.874 |
| Future Promise | 0.055 | 0.254 | 0.05 | 0.235 | 0.06 | 0.26 | 0.518 |
| Gendered Language | 0.171 | 0.484 | 0.11 | 0.334 | 0.19 | 0.523 | 0.002 |
| Grindstone | 1.812 | 1.744 | 1.92 | 1.817 | 1.78 | 1.718 | 0.134 |
| Recruitability | 0.004 | 0.063 | 0 | 0.068 | 0 | 0.062 | 0.839 |
| Humble | 0.099 | 0.326 | 0.06 | 0.255 | 0.11 | 0.346 | 0.004 |
| Leadership | 0.652 | 1.051 | 0.74 | 1.22 | 0.62 | 0.986 | 0.036 |
| Likeability | 0.239 | 0.487 | 0.21 | 0.439 | 0.25 | 0.502 | 0.127 |
| Maturity | 0.246 | 0.529 | 0.24 | 0.54 | 0.25 | 0.526 | 0.716 |
| Physical Description/Dress | 0.047 | 0.307 | 0.04 | 0.308 | 0.05 | 0.307 | 0.661 |
| Professionalism | 0.308 | 0.653 | 0.24 | 0.58 | 0.33 | 0.674 | 0.007 |
| Research | 3.242 | 3.849 | 3.32 | 3.711 | 3.22 | 3.896 | 0.615 |
| Respect | 0.237 | 0.515 | 0.23 | 0.525 | 0.24 | 0.512 | 0.855 |
| Personality | 0.43 | 0.761 | 0.35 | 0.703 | 0.46 | 0.778 | 0.011 |
| Superlatives | 3.607 | 2.787 | 3.6 | 2.559 | 3.61 | 2.861 | 0.946 |
| Take Charge | 0.064 | 0.254 | 0.08 | 0.273 | 0.06 | 0.248 | 0.277 |
| Teaching | 0.832 | 1.183 | 0.87 | 1.108 | 0.82 | 1.207 | 0.443 |
| Teamwork | 0.671 | 1.103 | 0.64 | 1.12 | 0.68 | 1.097 | 0.439 |
| Technical Skills | 0.658 | 0.822 | 0.7 | 0.83 | 0.64 | 0.819 | 0.243 |
| Trustworthy | 0.094 | 0.327 | 0.08 | 0.273 | 0.1 | 0.344 | 0.17 |
| Integrity/Honesty | 0.208 | 0.518 | 0.15 | 0.437 | 0.23 | 0.542 | 0.006 |
| Work Ethic | 0.356 | 0.592 | 0.32 | 0.558 | 0.37 | 0.603 | 0.202 |

Supplemental Table 5. Mean counts of statistically significant individual terms (p<0.05), by applicant gender

| **Stem Word** | **Overall avg** | **STD** | **Female avg** | **Female STD** | **Male avg** | **Male STD** | **p-value** | **category** |
| --- | --- | --- | --- | --- | --- | --- | --- | --- |
| man | 0.125 | 0.42 | 0.016 | 0.126 | 0.162 | 0.475 | 0 | ['Gendered Language'] |
| woman | 0.022 | 0.158 | 0.08 | 0.296 | 0.002 | 0.048 | 0 | ['Gendered Language'] |
| wife | 0.02 | 0.145 | 0 | 0 | 0.027 | 0.167 | 0.001 | ['Family'] |
| leadership | 0.252 | 0.594 | 0.33 | 0.714 | 0.226 | 0.545 | 0.002 | ['Agency', 'Leadership'] |
| gentleman | 0.016 | 0.126 | 0 | 0 | 0.022 | 0.146 | 0.002 | ['Gendered Language'] |
| husband | 0.013 | 0.115 | 0.027 | 0.164 | 0.008 | 0.092 | 0.003 | ['Family'] |
| introvert | 0.002 | 0.042 | 0.007 | 0.083 | 0 | 0 | 0.003 | ['Personality', 'Personality'] |
| profession | 0.346 | 0.697 | 0.268 | 0.638 | 0.372 | 0.713 | 0.007 | ['Professionalism', 'Professionalism'] |
| master | 0.113 | 0.349 | 0.151 | 0.406 | 0.1 | 0.327 | 0.008 | ['Agency', 'Agency'] |
| mentor | 0.201 | 0.514 | 0.256 | 0.608 | 0.182 | 0.477 | 0.009 | ['Teaching'] |
| seriou | 0.033 | 0.192 | 0.014 | 0.117 | 0.04 | 0.212 | 0.013 | ['Agency'] |
| abl | 0.385 | 0.769 | 0.465 | 0.825 | 0.358 | 0.748 | 0.013 | ['Technical Ability', 'Technical Ability'] |
| sympathi | 0.001 | 0.034 | 0.005 | 0.068 | 0 | 0 | 0.015 | ['Communality'] |
| ladi | 0.005 | 0.068 | 0.011 | 0.106 | 0.002 | 0.048 | 0.015 | ['Gendered Language'] |
| passiv | 0.003 | 0.064 | 0.009 | 0.117 | 0.001 | 0.028 | 0.017 | ['Communality'] |
| public | 0.41 | 0.72 | 0.481 | 0.797 | 0.386 | 0.69 | 0.018 | ['Reseach', 'Reseach'] |
| journal | 0.153 | 0.514 | 0.103 | 0.424 | 0.17 | 0.539 | 0.018 | ['Reseach', 'Reseach'] |
| scholarship | 0.099 | 0.398 | 0.137 | 0.469 | 0.086 | 0.37 | 0.019 | ['Academic Background/Awards/Scholarship'] |
| nice | 0.025 | 0.163 | 0.009 | 0.095 | 0.03 | 0.18 | 0.02 | ['Likeability'] |
| humbl | 0.062 | 0.246 | 0.039 | 0.205 | 0.069 | 0.257 | 0.024 | ['Humble'] |
| singl | 0.024 | 0.158 | 0.039 | 0.194 | 0.019 | 0.143 | 0.025 | ['Family'] |
| coordin | 0.06 | 0.294 | 0.087 | 0.414 | 0.051 | 0.24 | 0.027 | ['Agency'] |
| grant | 0.048 | 0.278 | 0.073 | 0.417 | 0.04 | 0.212 | 0.032 | ['Reseach', 'Reseach'] |
| cheer | 0.005 | 0.072 | 0.011 | 0.106 | 0.003 | 0.056 | 0.036 | ['Communality', 'Likeability'] |
| care | 1.281 | 1.451 | 1.407 | 1.465 | 1.239 | 1.445 | 0.036 | ['Communality', 'Communality', 'Grindstone'] |
| present | 0.661 | 0.954 | 0.744 | 0.976 | 0.633 | 0.945 | 0.036 | ['Reseach'] |
| mother | 0.02 | 0.164 | 0.034 | 0.217 | 0.015 | 0.141 | 0.037 | ['Family'] |
| step | 0.105 | 0.425 | 0.069 | 0.287 | 0.117 | 0.462 | 0.038 | ['Academic Background/Awards/Scholarship'] |
| compassion | 0.08 | 0.283 | 0.103 | 0.326 | 0.072 | 0.267 | 0.047 | ['Communality'] |
| fund of knowledg | 0.135 | 0.359 | 0.105 | 0.322 | 0.144 | 0.371 | 0.049 | ['Clinical Skills/Fund of Knowledge'] |

Supplemental Table 6. Letter theme by letter writer gender

| **Variable** | **Letters by women writers, Mean (STD), n= 175** | **Letters by men writers, Mean (STD), n= 1532** | **p-value** |
| --- | --- | --- | --- |
| Agency | 4.1 (3.0) | 3.7 (2.8) | 0.0330 |
| Communality | 3.2 (2.2) | 3.1 (2.4) | 0.1473 |
| Academic background, Awards, Scholarship | 2.4 (3.5) | 2.1 (3.2) | 0.0612 |
| Aptitude, Intelligence | 1.1 (1.8) | 0.8 (1.3) | 0.0541 |
| Clinical skills, Fund of Knowledge | 0.9 (1.7) | 0.5 (0.8) | 0.3110 |
| Communication | 0.2 (0.5) | 0.1 (0.4) | 0.3309 |
| Confidence | 0.4 (0.7) | 0.4 (0.7) | 0.2793 |
| Family | 0.5 (1.2) | 0.5 (1.2) | 0.3471 |
| Hobbies | 0.05 (0.3) | 0.02 (0.2) | 0.2414 |
| Future promise | 0.04 (0.2) | 0.06 (0.3) | 0.5210 |
| Gendered language | 0.2 (0.5) | 0.2 (0.5) | 0.9695 |
| Grindstone | 2.1 (1.9) | 1.8 (1.7) | 0.0362 |
| Humble | 0.1 (0.4) | 0.1 (0.3) | 0.5372 |
| Leadership | 0.9 (1.2) | 0.6 (1.0) | 0.0006 |
| Likeability | 0.3 (0.5) | 0.2 (0.5) | 0.0116 |
| Maturity, Emotional intelligence | 0.3 (0.5) | 0.2 (0.5) | 0.1135 |
| Physical description | 0.06 (0.4) | 0.05 (0.3) | 0.6750 |
| Professionalism | 0.3 (0.6) | 0.3 (0.6) | 0.8434 |
| Research | 4.0 (4.6) | 3.1 (3.7) | 0.0156 |
| Respect | 0.4 (0.7) | 0.2 (0.5) | <0.0001 |
| Retention, Recruitment | 0.006 (0.08) | 0.004 (0.06) | 0.7250 |
| Personality | 0.5 (0.8) | 0.4 (0.8) | 0.2331 |
| Superlatives | 3.7 (2.7) | 3.5 (2.8) | 0.4166 |
| Take charge | 0.1 (0.3) | 0.06 (0.2) | 0.0071 |
| Teaching | 1.1 (1.2) | 0.8 (1.2) | <0.0001 |
| Teamwork | 0.8 (1.1) | 0.7 (1.1) | 0.0119 |
| Technical Ability / Skills | 0.7 (0.9) | 0.7 (0.8) | 0.8285 |
| Trustworthy | 0.1 (0.3) | 0.1 (0.3) | 0.7579 |
| Integrity, Honesty | 0.2 (0.4) | 0.2 (0.5) | 0.6321 |
| Work ethic | 0.4 (0.6) | 0.4 (0.6) | 0.6028 |
